# Impact of Fluid Dynamics on the Viability and Differentiation Capacity of 3D-Cultured Jaw Periosteal Cells

**DOI:** 10.3390/ijms23094682

**Published:** 2022-04-23

**Authors:** Wanjing Cen, Suya Wang, Felix Umrath, Siegmar Reinert, Dorothea Alexander

**Affiliations:** 1 Department of Oral and Maxillofacial Surgery, University Hospital Tübingen, 72076 Tübingen, Germany; cenwanjingwj@gmail.com (W.C.); suyawang1227@gmail.com (S.W.); felix.umrath@med.uni-tuebingen.de (F.U.); siegmar.reinert@med.uni-tuebingen.de (S.R.); 2 Department of Orthopedic Surgery, University Hospital Tübingen, 72072 Tübingen, Germany

**Keywords:** perfusion bioreactor system, tangential and sigmoidal flow configuration, jaw periosteal cells, bioresorbable β-TCP scaffold, cell proliferation, osteogenic differentiation

## Abstract

Perfused bioreactor systems are considered to be a promising approach for the 3D culturing of stem cells by improving the quality of the tissue-engineered grafts in terms of better cell proliferation and deeper penetration of used scaffold materials. Our study aims to establish an optimal perfusion culture system for jaw periosteal cell (JPC)-seeded scaffolds. For this purpose, we used beta-tricalcium phosphate (β-TCP) scaffolds as a three-dimensional structure for cell growth and osteogenic differentiation. Experimental set-ups of tangential and sigmoidal fluid configurations with medium flow rates of 100 and 200 µL/min were applied within the perfusion system. Cell metabolic activities of 3D-cultured JPCs under dynamic conditions with flow rates of 100 and 200 µL/min were increased in the tendency after 1, and 3 days of culture, and were significantly increased after 5 days. Significantly higher cell densities were detected under the four perfused conditions compared to the static condition at day 5. However, cell metabolic and proliferation activity under dynamic conditions showed flow rate independency in our study. In this study, dynamic conditions increased the expression of osteogenic markers (ALPL, COL1A1, RUNX2, and OCN) compared to static conditions and the tangential configuration showed a stronger osteogenic effect than the sigmoidal flow configuration.

## 1. Introduction

Organ loss or failure due to tumor resection, severe trauma, or congenital defects, is one of the most frequent, destructive, and cost-intensive problems, especially with regard to the regeneration of large bone defects. Due to the limitation of spontaneous bone self-healing, regeneration of large bone defects is beyond the normal healing potential [1,2]. For these cases, the tissue engineering approach comprising the use of autologous stem cells and degradable biomaterials represents a suitable and very promising therapeutic option. For bone tissue engineering, 3D porous scaffold materials as supporting structures are usually combined with osteogenic progenitor cells [3,4,5,6,7,8]. Three-dimensional grafts used for transplantations, such as skin flaps, are usually thin due to the limited cell survival in the central area of thick grafts [9]. Most mammalian cells are susceptible to culturing and stimulation conditions. The growth of 3D cultured cells in the static culture environment is limited by insufficient transport of oxygen and nutrients, and removal of waste products and metabolites within the scaffolds [10]. Additionally, in vitro static culture without appropriate mechanical stimulation does not simulate the natural microenvironment of bone tissue resulting in non-functional, poorly cell-colonized constructs [9,11]. To avoid these limitations, bioreactor systems were developed in order to provide dynamic culture conditions and to improve the quality of the tissue-engineered grafts [12,13,14]. In a study by Bruder et al., the implantation of a hydroxyapatite/β-TCP scaffold containing mesenchymal stem cells in a rat femoral gap model showed a positive impact on bone tissue growth around the periphery but lacked mineralization in the central region of the implant [15]. Considering these issues, the quality of tissue-engineered bone grafts has to be improved in terms of cell growth and differentiation within the whole construct. It has been reported that dynamic conditions within bioreactors resulted in a high level of human mesenchymal stem cell viability allowing a maximum size of a half adult femur (∼200 cm^3^) [16]. Besides improved growth conditions, dynamic culture conditions stimulated stem cell differentiation by enhancing osteogenic gene expression, and consequently promoting tissue maturation within the bioreactor [12,16].

As developments progress, some of the bioreactors were put on the market and there are increasing reports of their clinical usage [17]. Different concepts of dynamic 3D bioreactors were developed in order to simulate the natural microenvironment within the bone tissue, for example, spinner flasks, rotating wall vessel constructs, perfusion bioreactors, and systems allowing mechanical or electromagnetic stimulation of cell/scaffold composites [18].

Beta-tricalcium phosphate (β-TCP) is one of the most used synthetic bone substitute materials in bone tissue engineering due to its good biocompatibility, high osteoconductive and osteoinductive properties. β-TCP is not soluble in physiological conditions, but can be resorbed by osteoclasts, leading to material dissolution and final replacement by new bone formation [19]. Marc Bohner and co-authors has documented the widespread interest in this material, reflected in more than 200 articles published yearly [20]. In previous studies, we used this material for biofunctionalization strategies in order to improve cell functions of jaw periosteal cells (JPCs) [7,21,22,23]. We have shown in numerous studies that JPCs represent a suitable stem cell source for the generation of bone tissue engineering (BTE) constructs which can be used without constraints for bone regeneration purposes in the oral and maxillofacial region [5,24,25].

In the present study, we aimed at establishing an optimal perfusion condition in order to create cell-rich bone-like scaffolds. For this purpose, we used a commercially available perfusion bioreactor for the cultivation of JPCs seeded on β-TCP scaffolds.

## 2. Results

### 2.1. Cell Proliferation within JPC-Seeded Scaffolds Cultured in the Perfusion System

**Figure 1:**
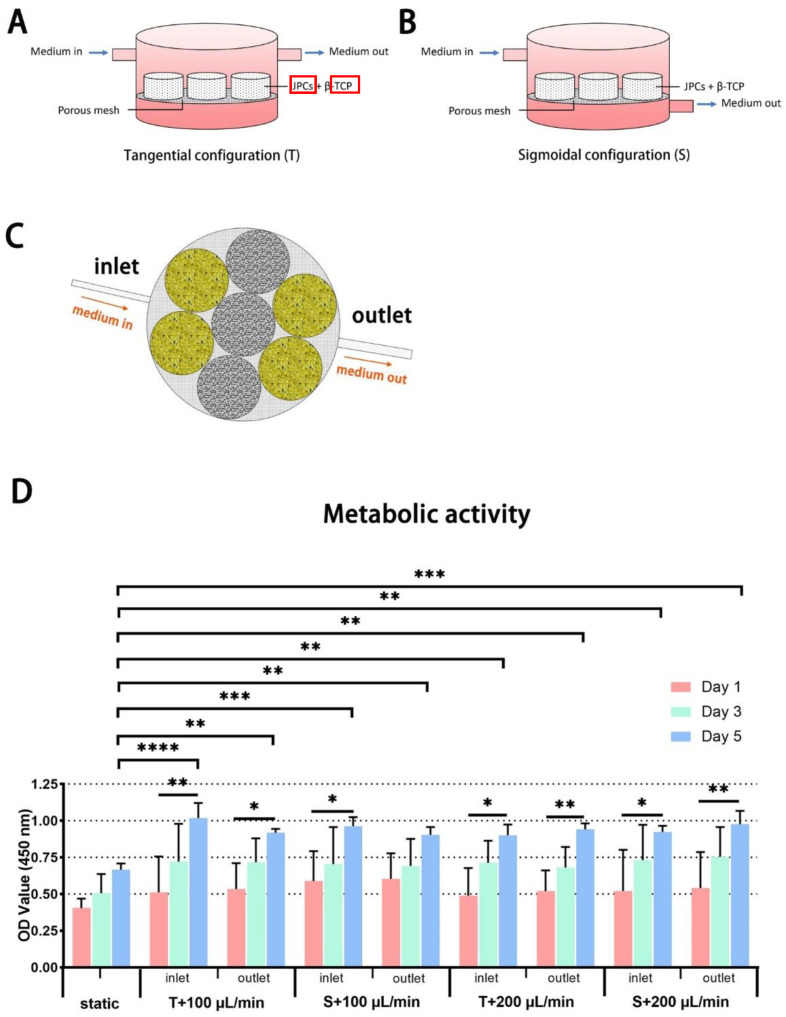
Schematic illustrations of (**A**) the tangential configuration and (**B**) the sigmoidal configuration. β-tricalcium phosphate (β-TCP) scaffolds were placed in the upper chamber of the bioreactor. (**C**) Position of scaffolds within the upper chamber of the bioreactor. (**D**) Metabolic activity of JPC-seeded scaffolds (yellow, positioned near the inlet and the outlet) under different culture conditions (static culture, tangential and sigmoidal configurations with flow rates of 100 and 200 µL/min) at day 1, 3 and 5 of in vitro cultivation. Optical density (OD) was measured at 450 nm and values are given as means ± standard deviation (SD). Results were compared using one-way ANOVA followed by Tukey’s multiple comparisons tests, the asterisk character reflects different *p*-values (* *p* < 0.05, ** *p* < 0.01, *** *p* < 0.001, **** *p* < 0.0001 (*n* = 3 donors)).

Cell proliferation was analyzed indirectly by measuring the metabolic activity of JPC-seeded scaffolds under the indicated configuration, flow rate and position within the bioreactor (Figure 1A–C). As shown in Figure 1D, significantly higher metabolic activities were detected within perfused scaffolds in comparison to static conditions at day 5. Compared to day 1, significantly higher metabolic activities were obtained at day 5 under most of the conditions, except under the static condition and sigmoidal configuration with a flow rate of 100 µL/min. No significant differences were detected among dynamically cultured JPC-seeded scaffolds under different flow configurations, different flow rates or different positions within the bioreactor.

### 2.2. Visualization and Quantitative Analysis of Cell Distribution within β-TCP Scaffolds Cultured under Static and Perfusion Conditions by Crystal Violet Staining

**Figure 2:**
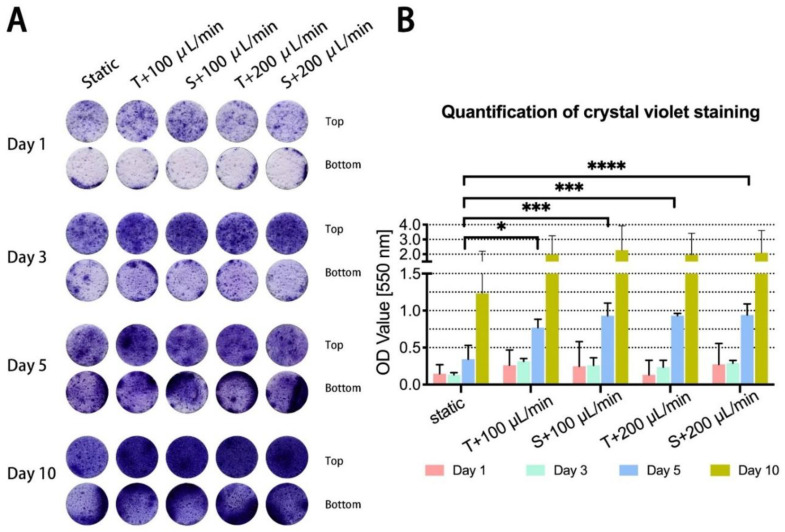
Crystal violet staining for the visualization and quantitative analysis of cell distribution within β-TCP scaffolds. (**A**) JPC-colonized scaffolds cultured under the indicated conditions (static culture, perfusion culture of tangential and sigmoidal configuration with flow rates of 100 and 200 µL/min) were stained with crystal violet dye at day 1, 3, 5 and 10 of in vitro cultivation. (**B**) Crystal violet staining was dissolved from stained scaffolds and optical density (OD) values were measured at a wavelength of 550 nm. The OD values are given as means ± SD and compared using one-way ANOVA followed by Tukey’s multiple comparisons tests (* *p* < 0.05, *** *p* < 0.001, **** *p* < 0.0001 (*n* = 3 donors)).

To visualize proliferation, density, and distribution of JPCs on β-TCP scaffolds, JPC-seeded scaffolds cultured under different conditions were stained with crystal violet, which binds to proteins and nucleic acids of cells. On day 1 some small crystal violet spots were observed on the top of scaffolds under all conditions while crystal violet plaques were detected only at the bottom edges (side that touches the ground of the well plate) (Figure 2A). During further cultivation, small crystal violet spots became larger and merged on day 3 and day 5. Bigger and deeper violet plaques were observed at the bottom of scaffolds under all conditions on day 5 in comparison to day 1 and 3. Homogenous deep violet staining was visible on the top of the scaffolds under perfused condition on day 10, while lighter violet staining was detected on the top of the scaffolds under static conditions. To better compare cell densities on the JPC-seeded scaffolds cultured under different conditions, crystal violet staining was quantified. According to the absorbance detected at 550 nm, significantly higher densities of JPCs were detected under the four perfused conditions in comparison to the static condition on day 5 (Figure 2B). JPC densities on scaffolds under perfused conditions were shown to be higher in the tendency compared to those detected under static condition on day 10.

### 2.3. Visualization of Cell Morphology on JPC-Seeded β-TCP Scaffolds and Quantification of Scaffold Porosity by Scanning Electron Microscopy

**Figure 3:**
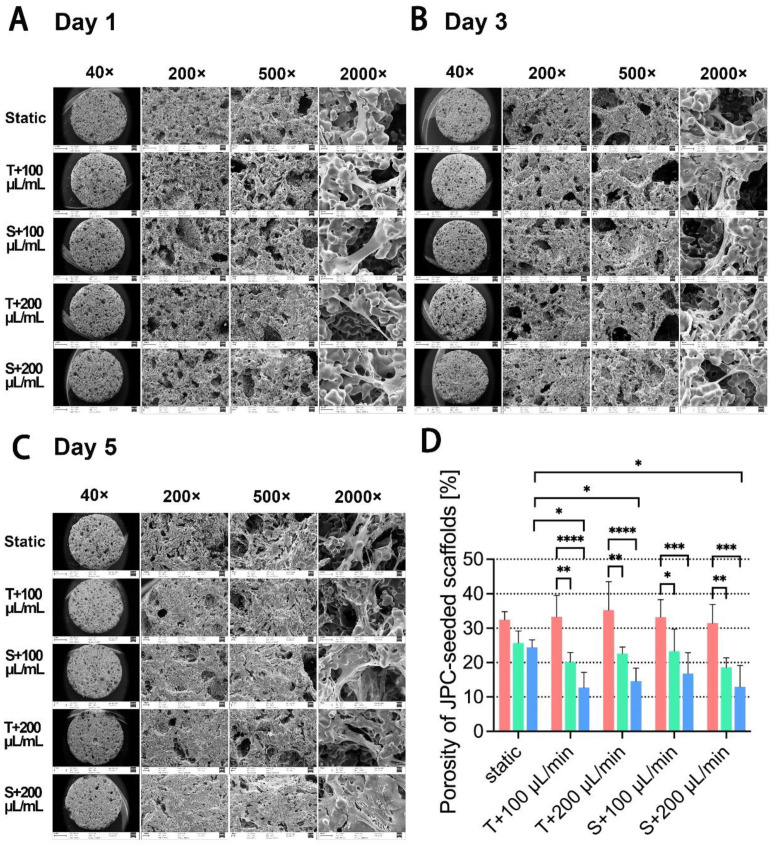
SEM micrographs of JPC-seeded scaffolds cultured for (**A**) 1, (**B**) 3 and (**C**) 5 days under the indicated conditions in different magnifications (40×, 200×, 500× and 2000×). Scale bars are 1 mm, 100 µm, 20 µm and 20 µm respectively. Representative images of three independent experiments (*n* = 3 donors with one experiment for each donor) are shown. (**D**) Pore areas in the 200× images were quantified with ImageJ software (red columns = day 1, green columns = day 3, blue columns = day 5). The porosity of JPCs-colonized scaffolds was calculated by the ratio of the total pore area to the whole area of the scaffolds. The ratios are given as means ± SD and compared using one-way ANOVA followed by Tukey’s multiple comparisons tests (* *p* < 0.05, ** *p* < 0.01, *** *p* < 0.001, **** *p* < 0.0001 (*n* = 3 donors)).

The morphology of the porous JPC-seeded β-TCP scaffolds was analyzed by scanning electron microscopy (SEM) after 1, 3 and 5 days of in vitro culture under the indicated conditions (Figure 3A–C). Differences in cell appearance were observed in different areas of the same scaffold surface, depending on the size of the pores into which the cells grew or which they spanned. In general, the JPCs appeared to preferentially grow inside small pores on the scaffold surface while the cells grew along the rim of the big pores as shown in images with 500-fold magnification. The SEM images revealed that JPCs spread over the pores of β-TCP scaffolds, fully expanded with a flattened morphology.

On day 1, the porosity of β-TCP scaffolds appeared to be high under all examined conditions under 200× and 500× magnifications and cell attachment was rarely observed (Figure 3A). During consecutive cell cultivation, the pore size and number decreased due to cell attachment and proliferation. At day 5, JPCs cultured under perfused conditions showed more homogeneous and deeper distribution within the scaffolds in comparison to the JPCs cultured under the static condition. As shown in Figure 3D, porosity of JPC-seeded scaffolds cultivated under perfused conditions decreased significantly at day 3 and 5 compared to the porosity observed at day 1. Significant lower porosity on perfused scaffolds (T + 100 µL/min, T + 200 µL/min and S + 200 µL/min perfused conditions) in comparison to static conditions were detected at day 5.

### 2.4. Visualization of Cell Density and Distribution within Scaffolds by Fluorescent Staining and Microscopy

**Figure 4:**
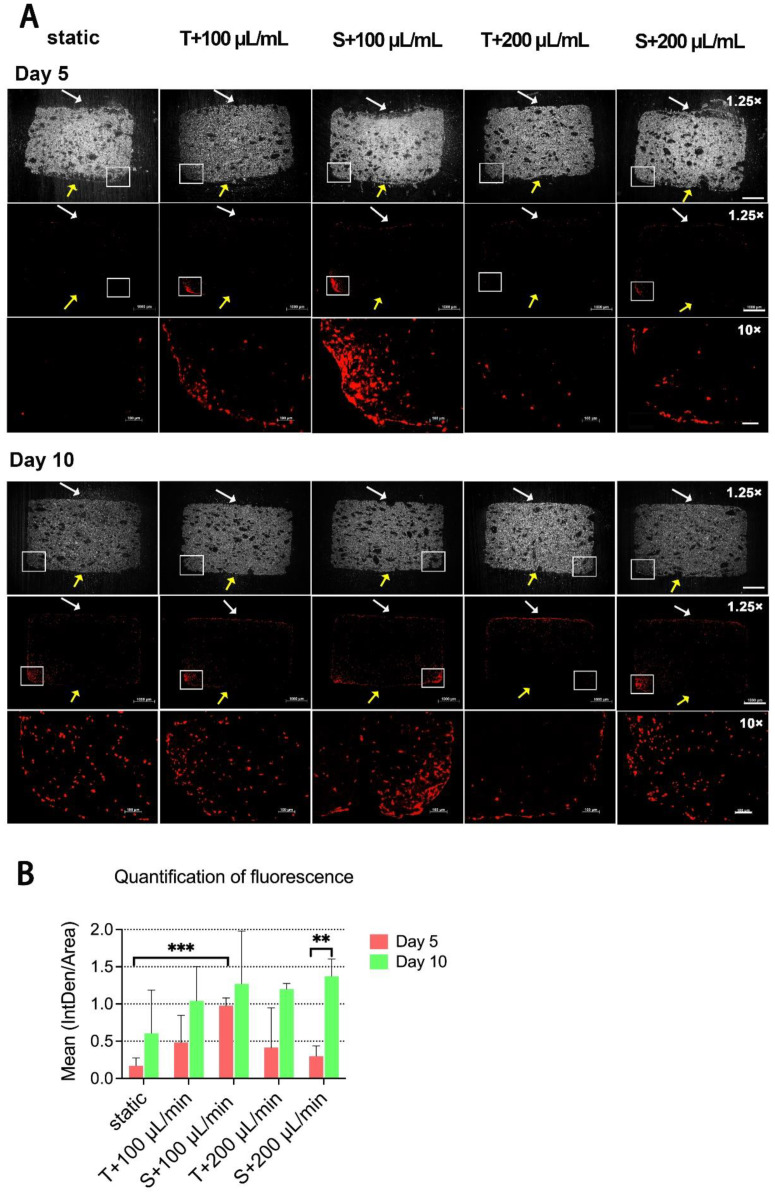
Visualization of cells in sections of PMMA-embedded scaffolds by Sytox orange nuclear staining. (**A**) Sytox orange staining was performed on sections of Technovit 9100 embedded JPC-seeded scaffolds that were cultured under the indicated conditions for 5 or 10 days. Bright field images with 1.25× magnification are given. White and yellow arrows point to the top and the bottom of the scaffolds respectively. Images taken with a 10-fold objective represent the area of rectangular box in images taken with a 1.25-fold objective. Scale bars represents 1 mm (1.25× magnification) and 100 µm (10× magnification) respectively. (**B**) Integrated densities (IntDen) in sections with 1.25 magnification at day 5 and 10 were quantified by the ImageJ software. Mean fluorescence intensities are shown as a ratio of IntDen to the total area of sections. Mean fluorescence intensity values are given as means ± SD and compared using one-way ANOVA followed by Tukey’s multiple comparisons tests (** *p* < 0.01, *** *p* < 0.001 (*n* = 3 donors)).

Since β-TCP scaffolds have a porous and brittle composition, we achieved the best experience with the polymethylmethacrylate (PMMA) embedding procedure. For further information about JPC distribution within the scaffolds under static or different perfusion conditions, sections of PMMA-embedded JPC-seeded scaffolds were stained by Sytox orange to visualize cell nuclei. The images of 1.25-fold magnification showed that JPCs were mainly located on the top (white arrows) and the bottom edge (rectangular boxes) of scaffolds after 5 days of culture. A higher fluorescence intensity was observed on the sections of the scaffolds under perfusion in comparison with scaffolds cultured under the static condition (Figure 4A). After 10 days of culture, cells appeared on both the surface and within the scaffolds under all conditions (Figure 4A). According to the quantification results of Sytox orange staining, means of red fluorescence under perfused conditions were higher than the ones obtained under static condition in the tendency at both day 5 and 10. Mean fluorescence under sigmoidal configuration with a flow rate of 100 μL/min was shown to be significantly higher compared to the detected mean fluorescence under static condition (Figure 4B).

### 2.5. Gene Expression Analyses of Osteogenic Markers in 3D-Cultured JPCs Growing under Perfusion Conditions

**Figure 5:**
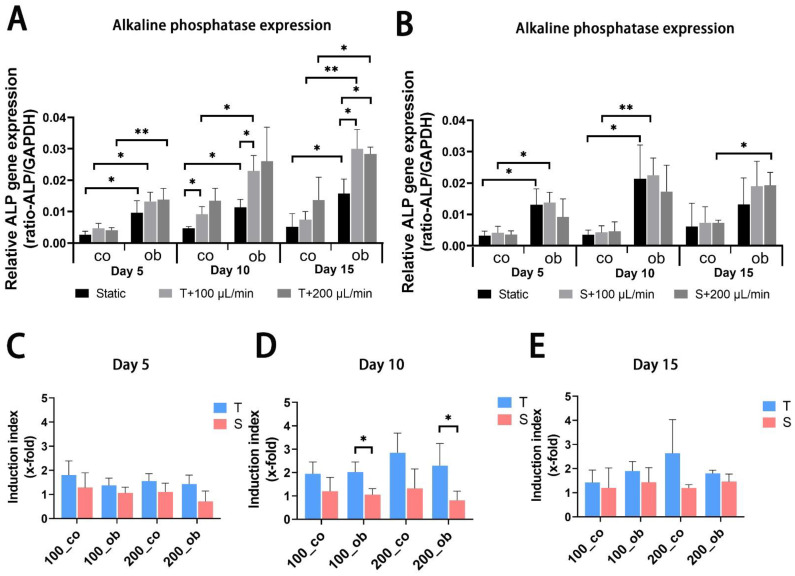
Gene expression levels of alkaline phosphatase (ALPL) in JPC-seeded β-TCP scaffolds after 5 days, 10 days and 15 days of osteogenic differentiation under static or perfusion conditions. mRNA expression levels under (**A**) tangential and (**B**) sigmoidal configuration were quantified and normalized to the housekeeping gene GAPDH. To calculate induction indices, gene expression levels were normalized to untreated (co) JPCs or osteogenically induced (ob) under static conditions. Induction indices of tangential (T) and sigmoidal (S) configuration after (**C**) 5 days, (**D**) 10 days and (**E**) 15 days of culture were calculated. The gene expression and induction values are given as means ± SD. Gene expression levels under different culture conditions (dynamic conditions and static condition) are compared using one-way ANOVA followed by Tukey’s multiple comparisons tests. To compare levels of osteogenic to control condition and induction values between T and S configuration condition, two-tailed Student’s *t*-test was used (* *p* < 0.05, ** *p* < 0.01, (*n* = 3 donors)).

**Figure 6:**
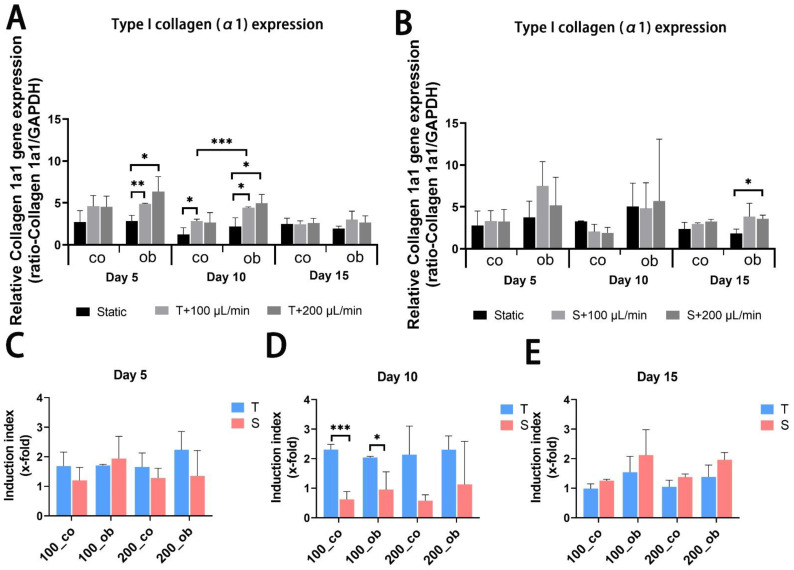
Gene expression levels of collagen 1α1 (COL1A1) in JPC-seeded β-TCP scaffolds after 5, 10 and 15 days of osteogenic differentiation under static or perfusion conditions. mRNA expression levels under (**A**) tangential and (**B**) sigmoidal configuration were quantified and normalized to the housekeeping gene GAPDH. To calculate induction indices, gene expression levels were normalized to untreated (co) or osteogenically induced (ob) JPCs under static condition. Induction indices of tangential (T) and sigmoidal (S) configuration after (**C**) 5 days, (**D**) 10 days and (**E**) 15 days of culture were calculated. The gene expression and induction values are given as means ± SD. Gene expression levels under different culture conditions (dynamic and static conditions) were compared using one-way ANOVA followed by Tukey’s multiple comparisons tests. For comparing levels of osteogenic condition to control and induction values between T and S, two-tailed Student’s *t*-test was used (* *p* < 0.05, ** *p* < 0.01, *** *p* < 0.001 (*n* = 3 donors)).

**Figure 7:**
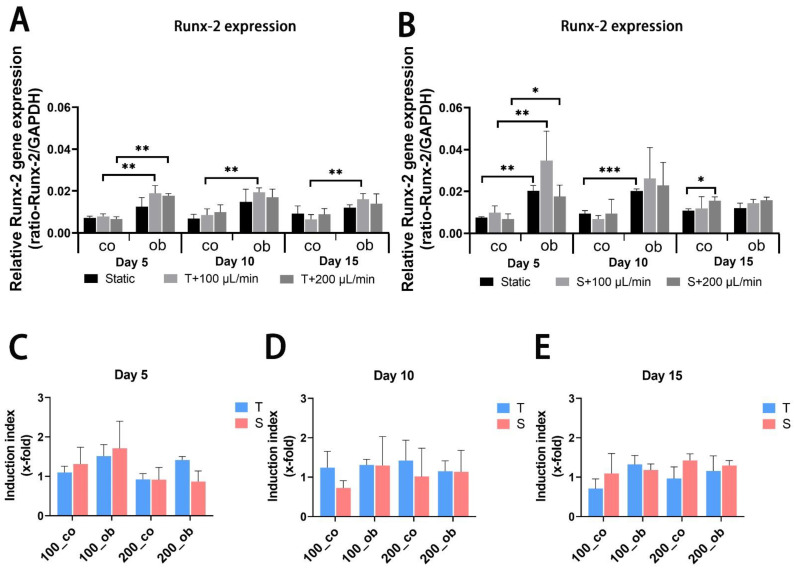
Gene expression levels of runt-related transcription factor 2 (RUNX2) in JPC-seeded β-TCP scaffolds after 5, 10 and 15 days of osteogenic differentiation under static or perfusion conditions. mRNA expression levels under (**A**) tangential and (**B**) sigmoidal configuration were quantified and normalized to the housekeeping gene GAPDH. To calculate induction indices, gene expression levels were normalized to untreated (co) or osteogenically induced (ob) JPCs under static condition. Induction indices of tangential (T) and sigmoidal (S) configuration after (**C**) 5 days, (**D**) 10 days and (**E**) 15 days of culture were calculated. The gene expression and induction values are given as means ± SD. Gene expression levels under different culture conditions (dynamic and static condition) are compared using one-way ANOVA followed by Tukey’s multiple comparisons tests. For comparing levels of osteogenic condition to control and induction values between T and S, two-tailed Student’s *t*-test is using (* *p* < 0.05, ** *p* < 0.01, *** *p* < 0.001 (*n* = 3 donors)).

**Figure 8:**
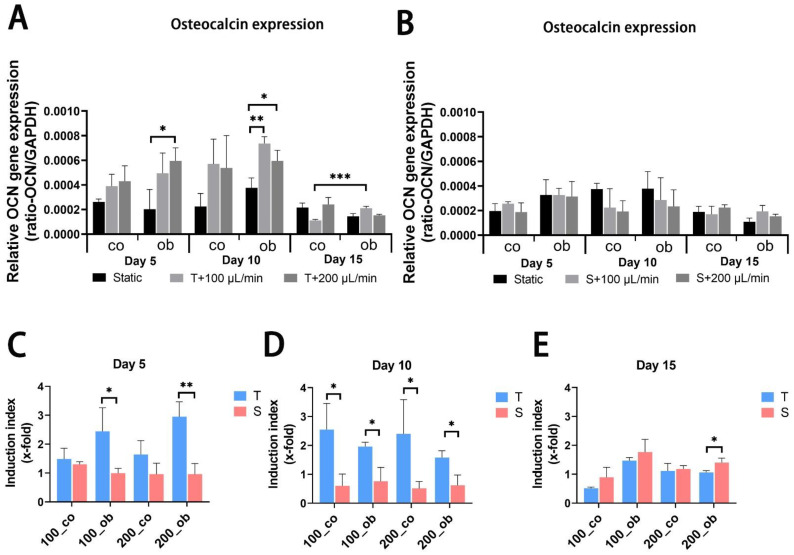
Relative gene expression levels of osteocalcin (OCN) in JPCs cultured within β-TCP scaffolds after 5, 10 and 15 days of osteogenic differentiation under the indicated conditions. mRNA expression levels under (**A**) tangential and (**B**) sigmoidal configuration were quantified and normalized to the housekeeping gene GAPDH. To calculate induction indices, gene expression levels were normalized to untreated (co) or osteogenically induced (ob) JPCs under static condition. Induction indices of tangential (T) and sigmoidal (S) configuration after (**C**) 5 days, (**D**) 10 days and (**E**) 15 days of culture were calculated. The gene expression and induction values are given as means ± SD. Gene expression levels under different culture conditions (dynamic conditions and static condition) were compared using one-way ANOVA followed by Tukey’s multiple comparisons tests. For comparing levels of osteogenic condition to control and induction values between T and S, two-tailed Student’s *t*-test is using (* *p* < 0.05, ** *p* < 0.01, *** *p* < 0.001 (*n* = 3 donors)).

JPC-seeded scaffolds were cultured dynamically or statically in osteogenic media and gene expression analysis was performed after 5, 10 and 15 days of 3D culture. mRNA expression levels of alkaline phosphatase (ALPL) and RUNX family transcription factor 2 (RUNX2) were significantly upregulated after 5 days of osteogenic induction under all performed conditions, but no significant differences between perfused conditions and static control (Figure 5A,B and Figure 6A,B) were observed. Gene expression levels of collagen 1α1 (COL1A1) were significantly higher under tangential configuration independently of the flow rates at day 5 (Figure 7A,B). In the case of osteocalcin (OCN), expression levels were increased significantly only under tangential configuration with 200 μL/min compared to obtained levels under static conditions in osteogenic media at day 5 (Figure 8A,B). At day 10, significantly higher ALPL and COL1A1 expression levels under tangential configuration with a flow rate of 100 μL/min were detected compared to levels under static condition in both control and osteogenic media. Further, significantly higher expression levels of OCN were observed under tangential configuration compared to levels detected under static and osteogenic condition. At day 15, significantly higher ALPL expression was detected under tangential configurations in osteogenic medium. Higher expression of RUNX2 was observed under sigmoidal configuration with a flow rate of 200 μL/min in control media, while increased expression of COL1A1 was detected under sigmoidal configuration with a 200 μL/min flow rate after 15 days of osteogenic differentiation (Figure 5, Figure 6, Figure 7 and Figure 8).

## 3. Discussion

Since cell fate and function are susceptible to the cells’ microenvironment, control over the microenvironment is essential in order to regulate cellular activities and behavior [26]. Many studies have shown that fluidic bioreactors are useful to control the microenvironment and have a high impact on promoting cell proliferation and differentiation. Perfusion bioreactor systems have shown promise for the 3D cultivation of stem cells for bone regeneration [27]. Beta-tricalcium phosphate, the scaffold material we used in this study, is a resorbable material that is widely used in clinics as a synthetic bone substitute [28]. It can be biofunctionalized in order to improve functions of the colonizing cells, as we reported in a previous study [7]. The aim of the present study was to establish optimal perfused conditions for the cultivation of JPC-seeded beta-TCP scaffolds, and to compare cell behaviors/functions under different perfusion conditions in comparison to the static culture condition. Flow rates during perfusion have to be optimized, since cells can be damaged at high flow rates, or may not have sufficient nutrients and oxygen supply at low flow rates. In our study, cell metabolic activities were increased in the tendency under dynamic conditions with flow rates of 100 and 200 µL/min after day 1, and 3 of perfusion culture, and reached significantly higher values at day 5 compared to the static culture (Figure 1C). This result was confirmed by the crystal violet staining which showed similar results (Figure 2B) as detected by the colometric assay. The flow rates we used in this study were supposed to be in the range of values reported to promote osteogenic cell proliferation [3,4]. Cartmell et al. [3] reported that a flow rate of 1.0 mL/min led to significant cell death and lowering of flow rate resulted in increased numbers of viable MC3T3-E1 cells. Best results were achieved with a flow rate of 100 μL /min compared to 200 μL /min and to the static controls [3]. However, in our study, very similar cell metabolic activities were obtained under dynamic conditions independently of the used flow rate. An important difference to these studies might be the fact, that perfusion chambers used here were much bigger than the scaffolds, which allowed the medium flow to go around the scaffolds. As a result, we made the observation that cells did not grow into the scaffolds and did not homogeneously cover the scaffolds, as shown by the crystal violet and Sytox orange staining. The flow of our perfusion system exerted shear stress in an unidirectional laminar manner to the scaffold surface under both configuration types (Figure 1A,B). A previous study demonstrated that bone cells respond to fluid-generated shear stress by an increase in intracellular calcium, providing evidence that fluidic flow alone can stimulate bone cells [29]. In our study, we expected that the shear stress exerted by the fluidic flow onto the scaffolds in the same chamber depended on the distance between the scaffolds and the medium inlet. But as shown in Figure 1C, flow configuration made no difference in cell viability of 3D-cultured JPCs, implying that shear stress forces within the chamber were uniformly distributed or too little to direct cell proliferation. Since the amount of medium in the perfusion system was 200 times more than in the cell culture plate for static culture, enhanced cell proliferation on perfused scaffolds can be explained by better supply of nutrients and better transportation of metabolic waste. Cell distribution on the surface of scaffolds was initially determined by the seeding procedure/density in the 96-well plate outside the perfusion system, showing cell-rich distribution on the top and bottom edge of the scaffolds (Figure 2A and Figure 4A). Crystal violet staining and SEM images revealed higher cell densities on scaffolds cultured under dynamic conditions compared to the static controls during the analyzed culture time period. McCoy and co-authors summarized in his review article that cells attached flatly to the rim of the scaffold pores were more prone to active impact of perfusion compared to cells that bridged pores thereupon underwent cytoskeleton deformations due to resistance to flow [30]. We observed JPCs with a flat morphology attached to the side or the inside of the beta-TCP scaffold pores, in the perfusion experiments at day 1 by SEM. At day 5, cells were prone to bridge some of the scaffold pores because of increasing confluence under perfusion conditions resulting in significantly decreased porosity on the perfused scaffolds (Figure 3D). On one hand, achieving higher cell densities is desired. However, reduced scaffold porosity minimizes the nutrient transport and the stimulating effect on the cells growing within the scaffold by flow perfusion. Further experiments are needed to determine the cell viabilities within the scaffolds for long-term culture. Overall, the data suggested that the perfusion system promoted JPCs proliferation in beta-TCP scaffolds under tangential and sigmoidal configuration with flow rates of 100 and 200 μL/min, with no evidence pointing to different effects in different types of configurations or flow rates. 

Within the osteons, bone cells are exposed to interstitial fluid flow through the bone canaliculi, where they respond to changes in fluid flow shear stress controlling bone formation [31]. Additionally, progenitor cells can be affected in their differentiation by changes in hydrostatic pressure and shear stress within the bone marrow [32]. The perfusion system can also be used to mimic mechanical stimulation to the cells growing within the constructs to promote osteogenic differentiation and extracellular matrix production. In our study, the comparison of static and dynamic cultivation in terms of osteogenic gene expression (ALPL, COL1A1, RUNX2, and OCN) revealed that dynamic conditions obviously increased the expression of the analyzed osteogenic markers and the tangential flow configuration had stronger osteoinductive effect than the sigmoidal configuration. As an early marker of osteogenic differentiation [33,34], alkaline phosphatase is reported to be upregulated by fluidic shear stress both on mRNA and protein expression level in MC3T3-E1 cells [3,4], and human osteoblasts [6] as well as in human MSCs [8,35]. Cartmell and co-authors reported from increased ALPL gene expression levels at 200 μL/min compared to conditions under lower flow rates. In our perfusion system mRNA levels of ALPL significantly increased compared to static conditions, and higher ALPL levels were induced under tangential compared to sigmoidal configuration in the tendency and at significant level under osteogenic condition at day 10. However, no significant change was achieved at different flow rates (100 and/or 200 μL/min). Compared to results obtained under sigmoidal configuration (Figure 6D,E) the tangential configuration setting seemed to be also more effective in the upregulation of COL1A1 gene expression (Figure 6D,E). RUNX2 represents an essential transcription factor involved in osteoblastic differentiation and skeletal morphogenesis [36,37]. Induction of RUNX2 was detected in osteogenic samples compared to the untreated ones, but perfused configurations had no significant effect on it. Osteocalcin (OCN) expression was significantly upregulated in the tangential configuration setting at day 5 and 10 of perfusion culture (Figure 8A,C,D). 

Taken together, in terms of metabolic activity/proliferation and distribution within the β-TCP scaffolds, we detected significant differences compared to static conditions, but we did not detect any correlation to fluidic dynamics or to the scaffold position within the used bioreactor. The tangential flow configuration seemed to activate osteogenic gene expressions by JPCs at a higher extent than the sigmoidal configuration set-up.

## 4. Materials and Methods

### 4.1. Isolation and Expansion of Jaw Periosteal Cells (JPCs)

The jaw periosteal tissue of three donors was obtained during routine surgery after informed written consent (approval number 6182017BO2 from the local ethics committee). The tissue (≤1 cm^2^) was cut into small pieces and washed with Dulbecco’s phosphate buffered saline (DPBS, Lonza, Basel, Switzerland). Then the fragments were enzymatically digested by 1500 U/mL collagenase (Sigma-Aldrich, Darmstadt, Germany) in DMEM/F12 medium for 2 h at 37 °C. After digestion, the cells were centrifuged, and cultured with DMEM/F12, containing the GlutaMAX supplement (Thermo Fisher Scientific, Waltham, MA, USA), 10% fetal bovine serum (Sigma-Aldrich, Darmstadt, Germany), 1% penicillin/streptomycin (Lonza, Basel, Switzerland), and 1% amphotericin B (Biochron GmbH, Berlin, Germany) at 37 °C in a humidified incubator. After 2 weeks of culture, JPCs were harvested for further expansion. The JPCs derived from 3 donors (two donors were 74 and one donor was 80 years old) of passage 5 were used in the experiment and culture medium was changed every other day.

### 4.2. Cell Seeding of β-TCP Scaffolds

The β-TCP scaffolds (Curasan AG, Kleinostheim, Germany) were soaked in culture medium for 1 h in low binding polypropylene 96-well plates before cell seeding. The JPCs were detached from the culture flasks with TrypLE Express (Thermo Fisher Scientific, Waltham, MA, USA) after reaching 80% confluency and resuspended in culture medium at a concentration of 1 × 10^6^ cells/mL. The medium was aspirated from the scaffolds and 50 µL of cell suspension (5 × 10^4^ cells) per scaffold was added. After 2 h of incubation, additional 150 µL of culture medium was added to the cell-seeded scaffolds, resulting in 200 µL final volume. For osteogenic differentiation, JPC-seeded scaffolds were cultured with osteogenic (OB) medium containing DMEM/F12, 10% FBS, 1% penicillin/streptomycin, 1% amphotericin B, 100 µM ascorbic acid 2-phosphate, 10 mM β-glycerophosphate and 4 µM dexamethasone (Sigma-Aldrich, Darmstadt, Germany) for the indicated time periods.

### 4.3. Cultivation and Configuration of the Perfusion Bioreactor

The double flow bioreactor (LB2, IVTech S.r.l., Ospedaletto, Italy), contains two chambers, with two flow inputs and outputs respectively (Figure 1A,B). The JPC-seeded scaffolds were placed in the upper chamber of the bioreactor on a porous nylon mesh (Merck, Darmstadt, Germany) with a pore size of 100 µm. Different set-ups of tangential and sigmoidal configurations (Figure 1A,B) with flow rates of 100 and 200 µL/min were applied (T100, T200, S100, and S200). JPC-seeded scaffolds cultured in a 24-well plate under control (CO) or osteogenic (OB) medium were used as controls for static conditions.

### 4.4. Cell Metabolic Activity Assay

The JPC-seeded scaffolds were cultured within the bioreactor under the indicated flow rate and flow configuration. After 1, 3 and 5 days of culture, JPC-seeded scaffolds were placed in a 96-well plate and incubated with 20 µL substrate (EZ4U, Biozol, Eching, Germany) and 200 µL culture medium for 2.5 h at 37 °C in a humidified incubator. 150 µL mixture of substrate and culture medium was pipetted into a fresh well, and the absorbance at 450 nm was measured using a microplate reader (Biotek, Bad Friedrichshall, Germany).

### 4.5. Crystal Violet Staining and Quantification

JPC-seeded scaffolds cultured under different conditions were fixed with 4% formaldehyde (Otto Fischar GmbH, Saarbrücken, Germany) for 20 min and then washed with PBS twice. 0.1% crystal violet dye (Sigma, St. Louis, MO, USA) was used to stain the fixed scaffolds for 20 min at room temperature. The scaffolds were washed with distilled water overnight on a shaker and dried at room temperature. The dye which was bound by the scaffolds was dissolved in 200 µL methanol for 30 min on a shaker. 150 µL of eluant was pipetted to a fresh well, and absorbance was measured at a wavelength of 550 nm with a microplate reader (Biotek, Bad Friedrichshall, Germany).

### 4.6. Scanning Electron Microscope (SEM) Analysis of JPC-Seeded Scaffolds

The JPC-seeded scaffolds were fixed with 4% glutaraldehyde (Applichem, Darmstadt, Germany) in 0.1 M sodium cacodylate (Merck, Darmstadt, Germany) buffer for 30 min and washed twice with PBS. All scaffolds were dehydrated with an ascending ethanol series (50%, 70%, 80%, 90% and 100%), liquids were completely removed by the critical point drying method and sputtered with a thin gold/palladium layer. The surface of the gold/palladium coated scaffolds was visualized with a scanning electron microscope (Carl Zeiss, Oberkochen, Germany). Scaffold porosities were measured in the 200× images using the quantification tools of the ImageJ software 1.53.

### 4.7. Embedding and Microtome Sectioning of Embedded JPC-Seeded Scaffolds 

JPC-seeded scaffolds were fixed with 4% formaldehyde for 30 min, washed twice with PBS, and distilled water, respectively. Then, samples were dehydrated and degreased with an ascending ethanol series (70%, 80%, 96% and 100%) for 15 min, and xylene for 15 min, respectively. Afterwards, all samples were pre-infiltrated with acetone twice for 1h and incubated with solution A of Technovit 9100 (Kulzer, Wehrheim, Germany) overnight at −20 °C. The following day, scaffolds were embedded with nine parts of solution A and one part of solution B in a 5 mL syringe and incubated again at −20 °C overnight. Finally, the syringes containing the scaffolds were incubated at 37 °C in a water bath for 1 h. Embedded scaffolds were cut to 15 µm sections with a microtome before fluorescent cell labeling.

### 4.8. Fluorescent Cell Labeling with Sytox Orange

Microtome sections were stained with 1:1000 Sytox orange dye (Thermo Fisher Scientific, Waltham, MA, USA) in PBS for 15 min and washed with PBS. All slices were mounted with Fluoromount-G (Thermo Fisher Scientific, CA, USA) before visualization by a fluorescent microscope (Carl Zeiss, Oberkochen, Germany).

### 4.9. Gene Expression Analyses by Quantitative PCR 

The JPC-seeded scaffolds were placed in Lysing Matrix D microtubes with ceramic beads (MP Biomedicals, Irvine, CA, USA) and lysis buffer (Macherey-Nagel, Dueren, Germany), and shredded by a FastPrep-24 device (MP Biomedicals, Irvine, CA, USA). RNA isolation from the obtained lysates of JPC-seeded scaffolds was carried out using the NucleoSpin RNA Mini kit (Macherey-Nagel, Dueren, Germany) following the manufacturer’s instructions. After RNA quantification using the Nanodrop spectral photometer (Thermo Fisher Scientific, Waltham, USA), 200 ng of RNA was synthesized to cDNA using the SuperScript VILO kit (Thermo Fisher, Darmstadt, Germany) following the manufacturer’s instructions. mRNA transcription levels were quantified by a real-time LightCycler system (Roche Diagnostics, Mannheim, Germany). DNA Master SYBR Green 1 (Roche, Mannheim, Germany) and the primer kits (Search LC, Heidelberg, Germany) for the target genes (ALPL, COL1A1, RUNX2, OCN, GAPDH) were used for the PCR reactions. 35 cycles of amplification were carried out for each mRNA. The ratio of target gene copy numbers to those of the housekeeping gene (GAPDH) were calculated.

### 4.10. Statistical Analysis

Statistical analysis was conducted for three independent experiments using the GraphPad Prism 8.1.0 software (GraphPad, San Diego, CA, USA), and all the results were presented as means ± SD. All data were tested for normality using the Shapiro-Wilk test. Statistical analysis was performed for comparing results of different culture conditions (dynamic conditions and static condition) using one-way ANOVA followed by Tukey’s multiple comparisons tests, for comparing results of osteogenic condition to control using two-tailed Student’s *t*-test. *p* values ≤ 0.05 were considered significant.

## 5. Conclusions

In this study, we tested a perfusion system for the simultaneous cultivation of several JPC-seeded beta-TCP scaffolds, which promotes cell proliferation and enhances osteogenic differentiation. Perfusion conditions stimulated cell growth on β-TCP scaffolds independently of the flow configuration and applied flow rates. The tangential configuration of the bioreactor seemed to up-regulate JPC gene expressions at a higher extent than the sigmoidal set-up and seems to be more suitable for the used beta-TCP constructs.

## Data Availability

Obtained data for this study are available from the corresponding author on reasonable request.
